# Compensation Method for Missing and Misidentified Skeletons in Nursing Care Action Assessment by Improving Spatial Temporal Graph Convolutional Networks

**DOI:** 10.3390/bioengineering11020127

**Published:** 2024-01-29

**Authors:** Xin Han, Norihiro Nishida, Minoru Morita, Takashi Sakai, Zhongwei Jiang

**Affiliations:** 1 Faculty of Engineering, Yamaguchi University Graduate School of Sciences and Technology for Innovation, 2-16-1 Tokiwadai, Ube City 755-0097, Yamaguchi Prefecture, Japan; hxyamaguchiu@163.com (X.H.); mmorita@yamaguchi-u.ac.jp (M.M.); 2 Department of Orthopedic Surgery, Yamaguchi University Graduate School of Medicine, 1-1-1 Minamikogushi, Ube City 755-8505, Yamaguchi Prefecture, Japan; nishida3@yamaguchi-u.ac.jp (N.N.); cozy@yamaguchi-u.ac.jp (T.S.)

**Keywords:** work-related musculoskeletal disorders, ergonomic posture risk assessment, REBA, skeleton compensation, ST-GCN

## Abstract

With the increasing aging population, nursing care providers have been facing a substantial risk of work-related musculoskeletal disorders (WMSDs). Visual-based pose estimation methods, like OpenPose, are commonly used for ergonomic posture risk assessment. However, these methods face difficulty when identifying overlapping and interactive nursing tasks, resulting in missing and misidentified skeletons. To address this, we propose a skeleton compensation method using improved spatial temporal graph convolutional networks (ST-GCN), which integrates kinematic chain and action features to assess skeleton integrity and compensate for it. The results verified the effectiveness of our approach in optimizing skeletal loss and misidentification in nursing care tasks, leading to improved accuracy in calculating both skeleton joint angles and REBA scores. Moreover, comparative analysis against other skeleton compensation methods demonstrated the superior performance of our approach, achieving an 87.34% REBA accuracy score. Collectively, our method might hold promising potential for optimizing the skeleton loss and misidentification in nursing care tasks.

## 1. Introduction

The nursing industry has consistently exhibited a high prevalence of work-related musculoskeletal disorders (WMSDs) [1]. Among nursing professionals, the incidence of work-related musculoskeletal disorders is even more pronounced, particularly in rehabilitation and geriatric care settings, reaching a staggering 92% [2,3]. The most effective preventive approach lies in conducting ergonomic posture risk assessments for nursing personnel and promptly addressing high-risk postures through corrective measures [4,5].

The predominant methods for assessing ergonomic posture typically rely on field observation or video monitoring to measure joint angles. These joint angles are then utilized in scoring tools, such as the Rapid Upper Limb Assessment (RULA) [6] and Rapid Entire Body Assessment (REBA) [7], to determine the level of postural risk and guide the implementation of suitable intervention measures. Nevertheless, limitations exist when conducting posture assessments through field observation. Firstly, subjective judgments made by assessors are prone to biases influenced by viewing angles and fatigue [8,9]. Secondly, manual observation is time-consuming and inefficient. As a result, researchers have sought to develop machine-based automated assessment methods as a replacement for manual evaluation. Initially, some researchers employed contact-based sensors to capture human posture movements. While this method provides high accuracy and frequently serves as a validation benchmark for emerging recognition techniques [10,11], it requires a significant number of sensors, resulting in increased equipment costs and requiring extensive sensor calibration. Moreover, the use of sensors may impede the normal work of healthcare personnel [12,13]. In contrast, vision-based posture motion capture methods offer a non-contact approach that does not disrupt the tasks of healthcare providers [14]. Currently, this approach primarily relies on machine learning algorithms to recognize motion pose keypoints from images or videos [15,16], enabling the automatic calculation of the REBA posture score using these keypoints. Compared to the Microsoft Kinect camera [17] and various pose estimation networks (e.g., PoseNet [18], DensePose [19], HRNet [20]), OpenPose [21] is presently recognized as a widely utilized and reliable algorithm for human pose estimation, demonstrating stable skeletal tracking capabilities even in non-frontal views and video sequences.

We endeavored to incorporate OpenPose into the automatic REBA assessment of caregiver postures. However, our findings revealed significant discrepancies in the REBA scores and substantial fluctuations in joint angles. To explore the underlying reasons for this issue, we conducted an analysis of caregiver postures. The results revealed that when healthcare professionals were involved in posture estimation, the overlapping of limbs between nurses and patients not only led to the loss of skeletal information but also introduced complexities in distinguishing the skeletal structures of both parties. Consequently, this significantly compromised the accuracy of OpenPose in estimating caregiver postures, resulting in considerable fluctuations and errors in both REBA scores and joint angles. The simultaneous estimation of poses for multiple individuals presents inherent challenges that may compromise the accuracy of joint angle calculations and lead to inaccurate REBA scores, particularly in scenarios involving overlapping, occlusion, and intricate interactions among various body parts.

To improve the pose estimation deficiencies caused by body occlusion in nursing interactions, researchers have utilized the principle of left–right symmetry to compensate for missing skeleton keypoints [22]. However, this approach is applicable to pose captured from a frontal camera perspective, and deviations in camera angles result in corrected skeletal keypoints being positioned outside the body. To overcome this limitation, the Mask RCNN method has been utilized to detect human boundaries, thereby constraining the skeletal keypoints within the body’s boundaries [23]. Nonetheless, compensating for skeletal keypoints using the symmetry principle often encounters challenges when dealing with complex movements. To restore occluded keypoints, researchers have explored the utilization of unoccluded skeletal keypoints in a Euclidean distance matrix [24]. This skeleton compensation method has proven successful in mitigating skeletal occlusion issues. However, ignoring temporal attributes and their association with skeletal motion trends leads to disparities between the compensated skeleton and the action dynamics. Furthermore, certain approaches have introduced the concept of “Human Dynamics” [25], which predicts future body poses based on multiple frames in the current video, even in the absence of subsequent frames. This method has demonstrated remarkable effectiveness in compensating for missing skeletal keypoints. However, limitations still persist regarding skeletal misidentification.

To tackle the challenges of skeleton loss and misidentification caused by body contact in nursing tasks, we proposed an enhanced spatial temporal graph convolutional network (ST-GCN) method that incorporated action feature weighting for skeleton time series. Additionally, we introduced a skeleton discrimination method based on kinematic chains, which identified skeletal loss and misidentification by combining skeleton and action features. This information was then utilized to provide feedback to the skeleton interpolation compensation network and skeleton correction network, enabling the reconstruction of missing and misidentified skeletal structures. The following are the main contributions of this study:

(1) An improved ST-GCN framework is proposed for skeleton action prediction.

(2) A kinematic-chain-based method for missing and misidentified skeletons is proposed for skeleton compensation in scenes with limb overlapping.

(3) Our results illustrate that the skeleton compensation and correction methods can effectively improve the calculation accuracy of skeleton joint angles and REBA score.

## 2. Methods

### 2.1. Overview

**Figure 1:**
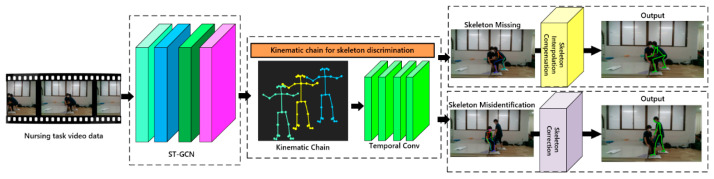
Overview of our skeleton compensation method.

In our study, we introduced a novel kinematic chain skeleton discrimination method to assess the integrity of the pose skeleton, distinguishing loss and misidentification. By analyzing the heterogeneity of action features obtained from the ST-GCN network and their corresponding skeleton mappings within a predefined temporal threshold, we identified instances of skeleton misidentification from a pose-based kinematic chain perspective. To optimize skeletal loss, we proposed a temporal-based skeleton interpolation compensation method. This involved utilizing temporal features, traversing complete skeletons preceding and subsequent to the temporal sequence, and employing interpolation algorithms to rectify missing skeleton data. In cases of skeleton misidentification, we presented a method to optimize action feature heterogeneity. This technique involved optimizing action features with lower weights within the predefined temporal range, compensating for gaps by utilizing consistent action features from previous and subsequent temporal sequences, and updating the corresponding skeletons mapped with the action features to rectify misidentification of the pose skeleton. The overview of our skeleton compensation method is shown in Figure 1. The following supporting information can be downloaded at: https://github.com/Nicxhan/Skeleton-compensation-and-correction (accessed on 1 January 2024).

### 2.2. ST-GCN

**Figure 2:**
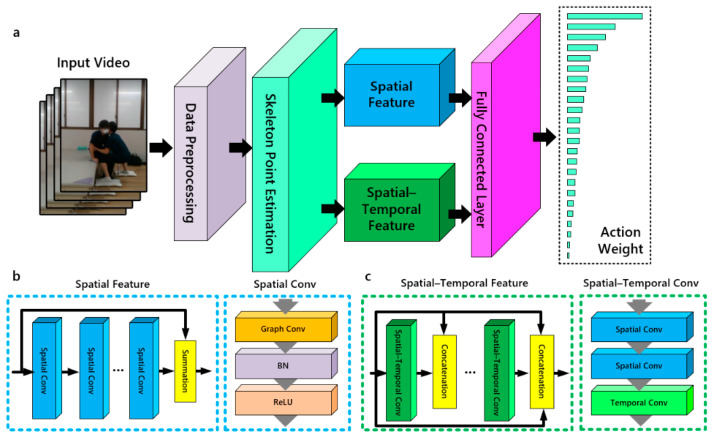
(**a**) Spatial temporal graph convolutional network structure. (**b**) Spatial Feature layer and Spatial Conv structure. (**c**) Spatial Temporal Feature layer and Spatial–Temporal Conv structure.

The ST-GCN has demonstrated its extraordinary ability to extract dynamic skeletal features from both spatial and temporal dimensions by capitalizing on a sequence of skeletal graphs [26]. Our adjusted ST-GCN structure comprises the spatial and spatial temporal feature layer (Figure 2a). Through the fusion of spatial temporal features of the skeleton, it enables the allocation of distinct action labels and weights to the temporal variations of skeletal features, redefining posture with actions.

The construction of the Spatial Feature layer entailed the integration of multiple Spatial Conv layers through residual structures. Each Spatial Conv layer was complemented by batch normalization (BN) and ReLU modules (Figure 2b), thereby bolstering the stability and facilitating the capture of intricate non-linear linkages among joints. The Spatial Feature layer aimed to discern the interconnected features that manifested between skeletal nodes and their neighboring counterparts, originating from the spatial information encapsulated within the pivotal nodes of the skeletal graph. Consequently, it exerted a discernible influence on the estimation of human poses by representing localized attributes of individual skeletal joints alongside the distinctive characteristics exhibited by adjacent nodes [27]. The Spatial–Temporal Feature layer, constructed by intricately interweaving multiple spatial temporal feature extraction units, manifested as a dense connection structure [28]. Encompassing a stack of Temporal Conv and Spatial Conv (Figure 2c), each Spatial–Temporal Conv aimed to extract motion trend features from skeletal joint nodes that exhibited correspondence across frames in the skeletal graph. This extraction process facilitated the depiction of motion trends between matched joint nodes in consecutive frames. By acquiring a comprehensive understanding of these features, the prediction of pose actions within the skeletal structure was enhanced.

### 2.3. Kinematic Chain for Skeleton Discrimination

The integration of spatial and temporal features within the label mapping framework enables the determination of action weights for postures, with the highest-weighted action label signifying each unique posture. To address challenges related to missing or misidentified skeletons in complex scenarios, we introduced a Kinematic Chain Skeleton Discrimination Network in the extra layer of the ST-GCN. This novel approach evaluated both skeletal pose completeness and the comparison of fused action weight features, distinct from prior research [29]. Anomalous action weights within a defined temporal sequence were identified as misidentified actions and skeletons, and corrective feedback was provided for both. Skeletal connections, denoting the links between adjacent keypoints in the human skeletal structure, form a 2 × *M* matrix *K*, where *M* represents the predefined number of skeletal keypoints. Matrix $\Psi =K^{T}K$ acts as a feature for discriminating skeletal integrity, with diagonal elements in Ψ representing squared joint lengths, while the remaining elements signify weighted angles between pairs of skeletal keypoints, serving as internal indicators. Inspired by kinematic chains, we introduced a temporal kinematic chain, defined as Equation (1).

(1) $$\Phi =K_{t+i}^{T}K_{t+i}-K_{t}^{T}K_{t}$$

where *i* represents the temporal interval between successive frames within the temporal kinematic chain. The diagonal elements within matrix Φ depict alterations in skeletal joint lengths, while the remaining elements signify changes in angles between pairs of skeletal keypoints.

We established the prediction of temporal kinematic chains by connecting the coordinates of skeletal keypoints, which were subsequently input into a Temporal Convolutional Network (TCN) to construct a posture discrimination network. This methodology not only accounted for the integrity of posture skeletons across frames but also ensured the coherence of weight variations in action feature changes across frames. It optimized abnormal action weights and provides feedback for skeleton compensation or correction. Building upon the framework of a Generative Adversarial Network [30], we constructed the posture discrimination network and employed this framework to generate regularization loss for pose estimation.

### 2.4. Skeleton Interpolation Compensation

**Figure 3:**
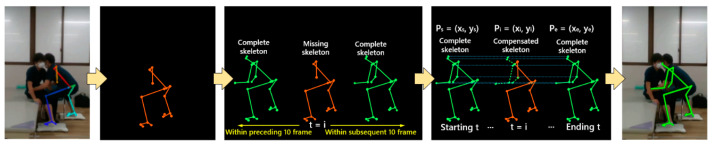
Skeleton compensation for missing frames (left to right: skeleton loss in OpenPose, missing skeleton frame, complete skeleton traverse, skeleton interpolation compensation, compensated skeleton).

In the case of missing skeleton states detected in the pose estimation results, the skeleton interpolation compensation network initiated the process by considering the current time sequence of the missing skeleton as the starting point. Subsequently, it traversed through the skeletal information of the preceding and succeeding time sequences to identify complete skeletons. In terms of temporal proximity to the missing skeleton, the nearest preceding and succeeding complete skeletons were chosen as references for interpolating the missing skeleton. Based on the spatial and temporal features offered by the complete skeletons, the linear interpolation algorithm was employed to fill in the missing skeletal keypoints. Simultaneously, the motion characteristics of the temporal sequence were taken into account to ensure alignment between the generated skeleton and the actual kinematic features, the process of skeleton compensation is depicted in Figure 3. To determine the temporal features within the interpolation compensation process, the traversal range for the preceding and succeeding temporal skeletons was set to 10 frames. This selection of a 10-frame range, sampled at a frequency of 50 Hz, provided the optimal interpolated data for motion skeleton interpolation [31].

Assuming that the motion velocity of skeletal keypoints remained independent and constant within the missing region, when there were *n* missing skeletal keypoints between the temporal sequences $P_{s}\left(x_{s},y_{s}\right),\;P_{e}\left(x_{e},y_{e}\right)$, $P_{s}$ and $P_{e}$ represented the starting and ending points of the complete skeletal information with a temporal distance of 10 frames, respectively. The missing point was denoted as $P_{1}\left(x_{1},\;y_{1}\right),\;P_{2}\left(x_{2},\;y_{2}\right),\;\ldots ,\;P_{n}\left(x_{n},\;y_{n}\right)$. The equation for computing the interpolated compensatory coordinates of the missing skeleton keypoints was determined by Equations (2)–(4).

(2) $$x_{i}=\left(1-t\right)x_{s}+tx_{e}$$

(3) $$y_{i}=\left(1-t\right)y_{s}+ty_{e}$$

(4) $$t=i/\left(n+1\right)\;\left(i=1,2,\ldots ,n\right)$$

### 2.5. Skeleton Correction

**Figure 4:**
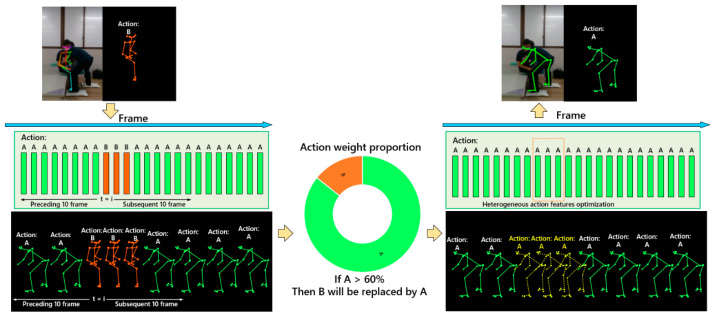
Skeleton correction for misidentified frames. It was accomplished by employing action features and weights when skeleton misidentification was detected, A and B represented the skeleton action features.

In the case of pose estimation results indicating skeletal misidentification states, we proposed a novel approach termed heterogeneous action feature optimization. By leveraging the inherent action features associated with each stage of the skeleton, we could rectify the misidentified skeleton by focusing on the correction of action features. The process of skeleton correction is depicted in Figure 4. The skeleton correction network commenced the process using the current time sequence of the misidentification skeleton as the starting point. It subsequently traversed the action features of the preceding and succeeding 10 frames within the temporal sequence. Following this, the weight proportions of the action features were calculated in the predefined time thresholds. For example, if the skeleton action features were denoted as A and B, within the specified time threshold, a comparison was made between the weights of action features A and B. Dominant action features were identified as those with a weight proportion exceeding 60%, while the remaining action features were considered heterogeneous. Consequently, the heterogeneous features were replaced with the dominant features, and the skeleton was accordingly updated. This approach effectively rectified the misidentified skeleton, demonstrating its efficacy in practice.

To prevent the disregard of preceding and succeeding frames due to estimation errors in the current frame, we incorporated the Kalman filtering algorithm to perform noise smoothing on the time series of coordinates for each skeletal point [32]. This procedure enhanced the congruity between the corrected skeleton and the actual movement. Assuming the independent calculation of each skeletal point, without considering skeletal constraints, we observed a natural correlation between the horizontal and vertical actions of the skeleton. Additionally, when disregarding action trends, the preceding and subsequent temporal states exhibited the same characteristics. Hence, Equations (5)–(9) were met.

(5) $${\hat{x}}_{k}^{-}=A{\hat{x}}_{k-1}+Bu_{k}$$

(6) $${\hat{P}}_{k}^{-}=AP_{k-1}A^{T}+Q$$

(7) $$K_{k}=\left(P_{k}^{-}C^{T}\right)/\left(CP_{k}^{-}C^{T}+R\right)$$

(8) $${\hat{x}}_{k}={\hat{x}}_{k}^{-}+K_{k}\left(y_{k}-C{\hat{x}}_{k}^{-}\right)$$

(9) $$P_{k}=\left(I-K_{k}C\right)P_{k}^{-}$$

where ${\hat{x}}_{k}$ and ${\hat{x}}_{k-1}$ represent the posterior state estimates of the skeleton points at time series *k* − 1 and *k*, respectively. ${\hat{x}}_{k}^{-}$ represents the prior state estimate of the skeleton point at time series *k*. $P_{k-1}$ and $P_{k}$ represent the posterior estimated covariance values at time series *k* − 1 and *k*, respectively. ${\hat{P}}_{k}^{-}$ represents the a priori estimated covariance value at time series k. C represents the transformation matrix from state variables to measured values. $y_{k}$ represents the input value. $K_{k}$ represents the Kalman coefficient. A represents the state transition matrix. B represents the control input matrix. Q represents the process excitation noise covariance value. R represents the measurement noise covariance value.

### 2.6. Study Design

The data used in this study was acquired by recruiting volunteers to simulate the task of patient transfer. The recruited volunteers had no history of musculoskeletal disorders in the past year. Volunteers were tasked with transferring the standard patient from the bed to the wheelchair. 

A single monocular RGB camera was employed for recording the nursing care task videos. A motion capture system comprising multiple inertial sensors was utilized to measure the angles of various joints in the body [33], with a high correlation observed between the results obtained from this system and those obtained from optical motion capture systems, making it suitable for joint angle measurement research. Additionally, inertial sensors possess strong occlusion resistance and find extensive application in fields like rehabilitation medicine and ergonomic analysis [34,35]. Hence, the joint angle measurements obtained from the inertial sensors can be employed as a ground truth value to assess the precision of visually based angle measurements [36]. 

Statistical analysis was conducted using SPSS v27 software (SPSS Inc., Chicago, IL, USA) and GraphPad Prism 9 (GraphPad Inc., San Diego, CA, USA). Paired *t*-tests were employed for paired continuous data, mean values and standard deviations were reported for all statistical tests. A *p*-value less than 0.05 was considered statistically significant.

### 2.7. Joint Angle and Scoring Tool

**Figure 5:**
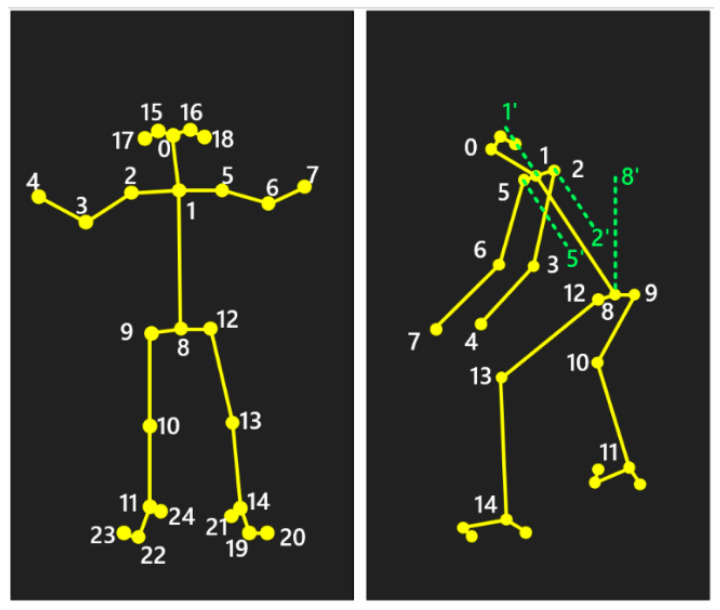
Pose estimation skeleton key points numbers. OpenPose detects 25 key skeletal points on the human body for joint construction and skeleton analysis. Numbers 0 to 24 represent different bone points.

The nursing task videos were processed by OpenPose and our method to predict the human body skeleton and compute the skeleton joint angles. A total of 25 skeletal keypoints were identified for each participant (Figure 5), and based on the scoring criteria of the REBA, a total of eight joint angles were calculated. The computation of joint angles and their corresponding skeletal keypoints were summarized in Table 1. Due to the wrist being in a nearly fixed position during the nursing tasks, the wrist angle was considered constant for the purpose of angle measurement and posture risk assessment in this study.

The REBA method was chosen as a tool for evaluating ergonomic risks in the workplace. Its objective was to swiftly assess the WMSD risk of postures to determine which work positions require additional attention and improvement, thereby reducing the risk of bodily discomfort and injury associated with work. The REBA algorithm involved evaluating the angle changes of key joints (trunk, neck, legs, upper arms, lower arms, wrists), external loads, and hand coupling capability. REBA scores range from 1 to 12, with higher scores indicating greater WMSD risk (Table 2).

### 2.8. Accuracy Verification

To validate the accuracy of our approach in posture risk assessment, a comparison was conducted among OpenPose, inertial sensors, and our method in terms of joint angles and REBA scores. The nursing task videos were separated into individual frames, and for each frame, the joint angles and REBA scores were calculated independently, as shown in Table 3. The mean absolute error (MAE) of the joint angles and the precision of the REBA scores were used to assess the performance of our method. The MAE measured the absolute difference between the joint angles computed by different methods. Although it did not distinguish between positive and negative errors, this value represented the actual magnitude of the error. The mathematical equation for MAE was determined by the Equations (10) and (11).

(10) $${MAE}_{1}=\left(\sum_{i=1}^{n}\left|A_{i}-A_{si}\right|\right)/n$$

(11) $${MAE}_{2}=\left(\sum_{i=1}^{n}\left|A_{oi}-A_{si}\right|\right)/n$$

where ${MAE}_{1}$ was measured by our method and the inertial sensors; ${MAE}_{2}$ was measured by OpenPose and the inertial sensors. Assuming the number of frames with consistent REBA scores between the inertial sensors and our method was denoted as *F_m_*, and the total number of frames was denoted as *F*, the REBA precision calculation was determined by Equation (12).

(12) $$Acc=F_{m}/F\times 100\%$$

## 3. Results

### 3.1. Missing and Misidentified Skeletons

**Figure 6:**
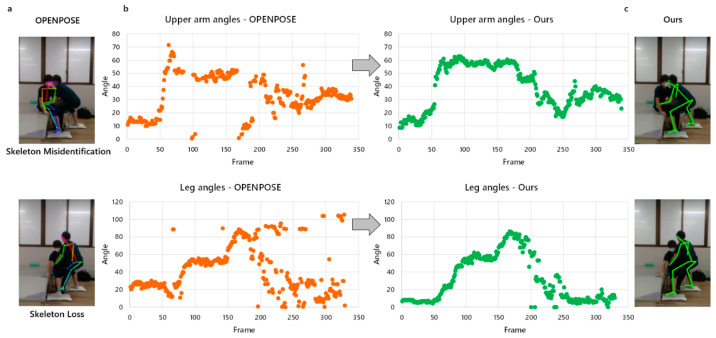
(**a**) The utilization of OpenPose for pose estimation in the nursing task gave rise to issues concerning missing and misidentified skeletons. (**b**) The variations in the angles of the upper arm and leg in the presence of skeleton loss and misidentification (Orange represents the angle data obtained by OpenPose) and subsequent skeleton compensation (Green represents the angle data obtained by our method). (**c**) The effect of our skeleton compensation method.

During the application of OpenPose for posture risk assessment in nursing tasks, notable challenges arise from complex interactions and overlapping body configurations between nurses and patients. These challenges often lead to incomplete or erroneous skeletal estimations, resulting in deviations and fluctuations in joint angles (Figure 6a). For instance, as depicted in Figure 6b, when a skeleton corresponding to the upper arm was misidentified, substantial fluctuations in the upper arm angle occurred, resulting in discontinuous states. In contrast, our method optimized the misidentification problem (Figure 6c), maintaining a stable and continuous state for the joint angles of the upper arm. Likewise, in scenarios where the skeleton was missing, such as the legs, there might be deviations or even a complete absence of leg angles. However, our method optimized the identification of the skeleton, achieving the continuity of leg angle measurements.

We compared the overall skeleton missing rate and misidentification rate for all frames (Table 4). The results revealed that our approach achieved a skeletal misidentification rate of 2.18%. Regarding the skeleton missing rate, except for the right lower arm (Lower arm-R) caused by limb occlusion, significant skeleton compensation effects were observed for all other missing skeletons. These outcomes highlighted the efficacy and potential of our approach in optimizing missing skeletons and misidentification the field of skeletal analysis.

### 3.2. Joint Angles Error

To assess the accuracy of our approach in measuring joint angles, we conducted a comparative analysis of angle errors among various methods. The analysis involved three distinct groups, each focused on evaluating the errors within a specific context. $E_{angle1}=A_{oi}-A_{si}$ represented the error between the joint angles obtained from OpenPose and the ground truth values; $E_{angle2}=A_{i}-A_{si}$ represented the error between our method and the ground truth values; $E_{angle3}=A_{i}-A_{oi}$ represented the error in joint angle errors between our method and OpenPose (Table 5).

We presented a detailed analysis of joint angle errors based on comprehensive experimental results (Table 5). When comparing joint angle errors between OpenPose and ground truth values (E_angle1_), all angles, except Trunk angles (*p*1 = 0.628), displayed significant statistical differences (*p*1 < 0.001), indicating substantial joint angle deviations. Conversely, our method exhibited minimal errors compared to ground truth values (E_angle2_), with significant statistical differences observed only in Upper arm-R (*p*2 = 0.025) and Lower arm-R (*p*2 = 0.006) joint angles. This highlighted the reliability of our method in calculating skeletal joint angles. Additionally, significant differences were found in joint angle errors (*p*3 < 0.001) between our method and OpenPose (E_angle3_), except for Trunk (*p*3 = 0.961) and Lower arm-R angles (*p*3 = 0.752), demonstrating the effectiveness of our approach in enhancing pose estimation accuracy and improving the precision of skeletal joint angle calculation.

**Figure 7:**
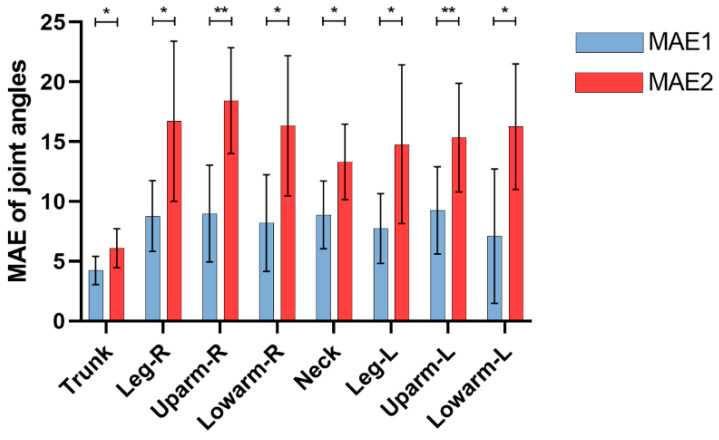
MAE of different joint angles. * *p* < 0.05, ** *p* < 0.01.

MAE was employed to evaluate the stability and accuracy of measuring joint angles. A smaller MAE value indicated better measurement accuracy. Our method consistently achieved an overall MAE (MAE1) below 10°, demonstrating superior accuracy in measuring joint angles (Figure 7). In contrast, OpenPose exhibited an MAE exceeding 10° for all joints, except the trunk, indicating significant error fluctuations. Both MAE1 and MAE2 showed statistically significant differences across all joint angles (*p* < 0.05). These discrepancies could be attributed to the skeleton loss and misidentification issues encountered in OpenPose during estimation of nursing care poses, resulting in frequent variations in angle differences and increased error fluctuation. In contrast, our proposed method addressed these challenges by optimizing skeleton loss and misidentification and reducing error fluctuations. This significantly enhanced the accuracy of joint angle calculations, as evidenced by the lower MAE values and reduced error fluctuations observed in Figure 7.

### 3.3. REBA Score Error

To verify the performance of our method in REBA scoring, we conducted a comparative analysis of the error in REBA scores among different skeletal joints. $E_{REBA1}=R_{oi}-R_{si}$ denoted the error between OpenPose and the ground truth values, while $E_{REBA2}=R_{i}-R_{si}$ signified the error between our method and the ground truth values. The results, in accordance with the REBA scoring rules, are presented in Table 6.

Based on the comprehensive results presented in Table 6, notable differences (*p* < 0.001) were observed in the joints scores and REBA scores between the OpenPose and the ground truth values (E_REBA1_), except for Trunk (*p* = 0.788) and Neck (*p* = 0.124). These observations indicated that the reliability of REBA scores derived from the OpenPose method for assessing nursing care task postures was suboptimal, with considerable deviations. Conversely, when considering the REBA scores obtained through our proposed method (E_REBA2_), a significant difference was only observed for the Lower arm-R score (*p* < 0.001) compared to the ground truth values, while no significant differences were detected for other joint scores. Moreover, the final REBA scores showed no significant discrepancy compared to the ground truth values (*p* = 0.373). These outcomes demonstrated that the REBA scores computed using our method closely aligned with the ground truth values, highlighting the substantial feasibility and reliability of our approach for assessing nursing task posture.

Moreover, to evaluate the effectiveness of our method in tackling the issues of skeleton loss and misidentification within nursing care task scenarios, we conducted a comprehensive performance comparison against several existing methods, including that of Tsai et al. [23], a left–right skeletal symmetry skeleton compensation method; Guo et al. [24], a Euclidean distance matrix skeleton compensation method; and Kanazawa et al. [25], a Human-Dynamics-based temporal skeleton compensation method. The evaluation metric employed for this analysis was the precision of REBA scores. To uphold the scientific integrity of the comparative results, all assessments of the methods were conducted using standardized hardware configurations and nursing care posture datasets. Nonetheless, it was vital to exercise caution when interpreting these findings, as discrepancies in algorithmic parameters and model metrics might introduce variations that require careful consideration [37]. The summarized results of this comparative evaluation can be found in Table 7.

The findings in Table 7 indicated that OpenPose achieved an accuracy exceeding 90% for specific skeletal joints, yet its final accuracy in REBA scoring remains at 58.33%. This was associated with the issues of skeleton loss and misidentification, which caused low accuracy of REBA. In contrast, our approach attained an accuracy of 87.34%, outperforming alternative methods and improving the skeleton loss and misidentification in nursing care tasks. Importantly, our method exhibited promising potential for pose assessment in interaction-based nursing tasks.

## 4. Discussion

### 4.1. Main Findings and Contributions

In this study, we identified concerning accuracy issues in the integration of OpenPose with the REBA assessment for nursing postures. This inadequacy stemmed from the inherent challenges posed by motion interactions and limb occlusions in nursing tasks, resulting in skeleton missing and misidentification in the OpenPose pose estimation. Consequently, these deviations and fluctuations in skeletal joint angles had a direct impact on the accuracy of REBA scoring. To address this problem, we have devised an innovative method that built upon the ST-GCN framework by incorporating action feature inverse skeleton compensation and correction. Hence, we enhanced the tracking of pose skeletons in scenarios involving overlapping bodies and interactive movements during nursing tasks. This improvement ensured the continuity and stability of skeletal joint angle calculations, ultimately resulting in an enhanced accuracy of REBA scoring.

To validate the reliability and feasibility of our proposed method, we conducted a comprehensive comparison of skeleton missing rate, skeleton misidentification rate, joint angles, REBA score, and REBA scoring accuracy. We have identified significant differences between the joint angles and scores obtained from OpenPose and the inertial sensors, primarily due to the influence of skeleton loss and misidentification. In contrast, our method yielded joint angles and scores that did not differ from the ground truth values, demonstrating the effectiveness of our approach in mitigating skeleton loss and misidentification challenges (Table 5 and Table 6). Furthermore, it was important to highlight that substantial angle errors were observed in the right upper and lower arm joints (Table 5, Upper arm-R (*p*2 = 0.025), Lower arm-R (*p*2 = 0.006)). This discrepancy could be attributed to the interaction between the arms and patients during the caregiving process, resulting in the loss of arm joint tracking features. It is important to note that such limitations are commonly encountered in vision-based pose estimation algorithms. It could be overcome by employing marker-based wearable sensor measurement methods, but the use of sensors itself may impede the normal work of healthcare personnel [12]. It seems that improving the performance of pose estimation algorithms is more convenient and effective [10]. While our method showed smaller error fluctuation (Figure 7), improvements could be made in the future studies, particularly in addressing errors related to the Leg, Upper arm, and Lower arm joints on the side that is occluded by the limb. These joints experience significant challenged in terms of skeleton loss during the pose estimation process within multi-person interaction nursing care tasks. Therefore, future research efforts should prioritize enhancing the recognition accuracy of these specific joints. 

While numerous studies have demonstrated the reliability of OpenPose in calculating joint angles for simple poses [38,39], its performance in complex scenarios involving overlapping bodies and interactions among multiple individuals remains suboptimal. Skeletal compensation methods that rely on left–right skeletal symmetry are often proved to be highly dependent on camera perspective settings [22]. Additionally, when employing Mask RCNN to confine the boundaries of compensated skeletal points in scenes with multiple individuals, the accuracy of pose skeleton estimation is not ideal enough [23]. Existing methods that compensate for occluded skeletons based on a Euclidean distance matrix [24] or that predict future pose skeletons using Human Dynamics [25] share a common limitation: they fail to address the problem of skeletal misidentification, leading to a uniform compensation approach for both correctly identified and misidentified skeletons. Consequently, the compensated skeletons fail to match the target pose skeleton, exacerbating differences in pose skeleton angles and REBA scores. Taking inspiration from skeleton kinematics, we proposed a novel skeleton discrimination method based on skeleton kinematic chains, which effectively distinguished different states of skeletal misidentification. Furthermore, we introduced a heterogeneous action feature optimization method that updated heterogeneous action features at the temporal sequences level. Leveraging the ST-GCN network’s ability to assign action labels to different temporal skeletons, we could focus on updating the action features to correct misidentified skeletons. Comparative analysis of the accuracy of REBA scores demonstrated the distinct advantages of our method compared to alternative approaches (Table 7).

Furthermore, the primary objective of this study was to conduct a comparative analysis between our method and the OpenPose in terms of the predictive accuracy of skeletal joint angles at the algorithmic level of 2D pose estimation. It is important to note that the REBA scoring criteria encompasses not only joint angle assessment but also incorporates additional scores for joint rotation and extra points. To ensure consistency across all methods, we manually defined the parameters for rotation and extra point interventions. While previous research has explored posture risk assessment based on monocular camera 3D pose estimation [40,41], achieving good recognition accuracy, it is essential to recognize the inherent limitations of 3D pose evaluation. The computational demands associated with 3D pose estimation make it less suitable for real-time pose estimation, and the reliance on depth cameras or specialized sensors to capture depth data introduces complexities in terms of hardware and data collection. In contrast, 2D pose estimation algorithms exhibit greater resilience to challenging conditions such as lighting variations and occlusions in comparison to their 3D counterparts. Significantly, most existing monocular camera 3D pose estimation techniques primarily focus on simple pose estimation scenarios, while the complexities arising from multi-person interactions and limb occlusions present more substantial obstacles for accurate 3D pose estimation. 

Collectively, our approach initially explored solutions for multi-person pose estimation from a 2D perspective before transitioning to 3D pose estimation research. The current research findings underscored the feasibility of our method, which might hold wide-ranging applicability in popular mobile devices or surveillance cameras through the utilization of lightweight models. Moreover, our method could be integrated into Internet of Things (IoT) devices equipped with RGB cameras, including smartphones and surveillance systems. Leveraging neural network models and image processing techniques, our method enables the inference of posture information, facilitating risk assessment and visual guidance for WMSDs associated with nursing postures. Looking ahead, the realization of an integrated intelligent nursing posture assessment system becomes a tangible possibility, driven by the advancements achieved through our method.

### 4.2. Limitations

It is important to acknowledge that our skeletal compensation and correction mechanisms rely on traversing temporal features over a span of 10 frames. Any instances of skeleton loss beyond this range might increase the skeleton miss rate of our method, resulting in our method’s REBA score accuracy being limited to 87.34%. As such, future investigations should focus on mitigating these limitations and exploring a suitable traversing temporal scope for improving accuracy. Furthermore, exploring the application of monocular camera 3D caregiving pose evaluation would be merited to improve the performance in the limb occlusion scenario, as investigating the effectiveness of 3D compared to 2D approaches would carry significant implications and contribute to the advancement of the field.

### 4.3. Directions for Further Research

In light of the demonstrable benefits associated with the capture of temporal features over a 10-frame interval in nursing care action interaction actions, the accuracy of skeleton compensation within this temporal range is influenced by the speed and complexity of these actions across diverse application scenarios. Consequently, it is imperative for future research to prioritize the investigation of pose actions’ intricacy and subsequently determine the optimal time span required to match these actions accurately. The development of a model that establishes the relationship between action complexity and time span would significantly enhance the efficiency and effectiveness of skeleton compensation, thereby unlocking the substantial potential for intelligent selection of time intervals in various pose estimation scenarios. Furthermore, augmenting the precision of monocular-camera-based 3D techniques in multi-person pose skeleton estimation is pivotal for improving the accuracy of caregiving posture assessment, particularly in scenarios involving rotational movements and changes in perspective. Exploring the integration of skeleton compensation and correction techniques derived from 2D approaches into 3D scenes represents a promising avenue for future research, as it addresses the challenge of compensating for skeleton occlusion during rotational maneuvers and visual alterations. Additionally, proactive exploration of the integration of our approach into Internet of Things (IoT) devices equipped with RGB cameras, such as smartphones and monitoring systems, holds substantial potential. Leveraging neural network models and image processing techniques to infer pose information can facilitate risk assessment and visual guidance pertaining to work-related musculoskeletal disorders (WMSDs), offering significant opportunities for the implementation of integrated intelligent pose assessment systems.

## 5. Conclusions

This study introduced an enhanced ST-GCN-based skeletal compensation method that effectively optimized skeletal occlusion and misidentification in nursing care tasks. Our approach integrated distinct action features and weights for posture skeletons, utilizing a skeletal discrimination network to evaluate skeleton integrity. To mitigate occlusion, we employed a skeletal interpolation compensation network that utilized adjacent temporal contexts. In instances of misidentification, a skeletal correction network optimized abnormal action features and updated skeletons accordingly. Our method improved joint angle calculations and enhanced the accuracy of REBA scores, which exhibited higher accuracy compared to the traditional OpenPose, achieving high precision in REBA scores for nursing task postures. Such improvements are crucial in mitigating the risk of WMSDs in the nursing profession.

## Figures and Tables

**Table 1:** Joint angles list.

Joint Angle	Involved Skeletal Points
Trunk flexion angle	∠1, 8, 8′
Neck flexion angle	∠0, 1, 1′
Left leg flexion angle	∠12, 13, 14
Right leg flexion angle	∠9, 10, 11
Left upper arm flexion angle	∠5′, 5, 6
Right upper arm flexion angle	∠2′, 2, 3
Left lower arm flexion angle	∠5, 6, 7
Right lower arm flexion angle	∠2, 3, 4

**Table 2:** REBA risk level list.

Action Level	REBA Score	Risk Level	Correction Suggestion
0	1	Negligible	None necessary
1	2–3	Low	Maybe necessary
2	4–7	Medium	Necessary
3	8–10	High	Necessary soon
4	11–15	Very high	Necessary now

**Table 3:** Accuracy calculation parameters.

	Nursing Task Video	Frame 1	Frame 2	Frame i	Frame n
OpenPose	Joint angle	A_o1_	A_o2_	A_oi_	A_on_
	REBA	R_o1_	R_o2_	R_oi_	R_on_
Inertial sensors	Joint angle	A_s1_	A_s2_	A_si_	A_sn_
	REBA	R_s1_	R_s2_	R_si_	R_sn_
Ours	Joint angle	A_1_	A_2_	A_i_	A_n_
	REBA	R_1_	R_2_	R_i_	R_n_
Accuracy	Joint angle error	[A_o1_, A_s1_, A_1_]	[A_o2_, A_s2_, A_2_]	[A_oi_, A_si_, A_i_]	[A_on_, A_sn_, A_n_]
	REBA score error	[R_o1_, R_s1_, R_1_]	[R_o2_, R_s2_, R_2_]	[R_oi_, R_si_, R_i_]	[R_on_, R_sn_, R_n_]

**Table 4:** Overall skeleton missing rate and misidentification rate for all frames.

Joints	Skeleton Missing Rate		Skeleton Misidentification Rate	
	OpenPose	Ours	OpenPose	Ours
Trunk	0.18%	0.07%	20.60%	2.18%
Leg-R	16.79%	5.96%		
Upper arm-R	22.42%	10.36%		
Lower arm-R	64.68%	51.67%		
Neck	22.06%	7.01%		
Leg-L	8.47%	1.78%		
Upper arm-L	11.19%	0.29%		
Lower arm-L	12.75%	0.58%		

**Table 5:** Errors between different joint angles.

Joints	E_angle1_ (N = 8)	*p*-Value *p*1	E_angle2_ (N = 8)	*p*-Value *p*2	E_angle3_ (N = 8)	*p*-Value *p*3
Trunk	−0.166 ± 18.526	*p* = 0.628	−0.019 ± 2.345	*p* = 0.659	−0.017 ± 18.800	*p* = 0.961
Leg-R	3.880 ± 18.591	*p* < 0.001	−0.060 ± 2.324	*p* = 0.160	0.882 ± 6.090	*p* < 0.001
Upper arm-R	3.145 ± 10.742	*p* < 0.001	−0.186 ± 4.475	*p* = 0.025	0.755 ± 10.136	*p* < 0.001
Lower arm-R	3.969 ± 30.840	*p* < 0.001	−0.226 ± 4.427	*p* = 0.006	−0.108 ± 18.481	*p* = 0.752
Neck	−1.956 ± 14.891	*p* < 0.001	−0.072 ± 2.281	*p* = 0.087	1.963 ± 14.436	*p* < 0.001
Leg-L	−1.069 ± 7.174	*p* < 0.001	−0.125 ± 4.512	*p* = 0.134	−4.098 ± 30.771	*p* < 0.001
Upper arm-L	−1.014 ± 10.605	*p* < 0.001	−0.059 ± 2.292	*p* = 0.165	0.773 ± 9.903	*P* < 0.001
Lower arm-L	2.473 ± 27.971	*p* < 0.001	0.006 ± 4.586	*p* = 0.942	−3.001 ± 27.793	*p* < 0.001

**Table 6:** Errors between joint angle score and REBA score.

Joints	EREBA1 (N = 8)	*p*-Value	EREBA2 (N = 8)	*p*-Value
Trunk	−0.001 ± 0.207	*p* = 0.788	0 ± 0.159	*p* = 1
Leg-R	0.255 ± 0.568	*p* < 0.001	0.015 ± 0.465	*p* = 0.066
Upper arm-R	−0.176 ± 0.644	*p* < 0.001	−0.005 ± 0.302	*p* = 0.296
Lower arm-R	−0.154 ± 0.635	*p* < 0.001	0.235 ± 0.448	*p* < 0.001
Neck	0.003 ± 0.132	*p* = 0.124	−0.003 ± 0.395	*p* = 0.638
Leg-L	−0.027 ± 0.282	*p* < 0.001	0.012 ± 0.506	*p* = 0.186
Upper arm-L	0.013 ± 0.282	*p* = 0.013	0.001 ± 0.186	*p* = 0.619
Lower arm-L	0.098 ± 0.309	*p* < 0.001	0.234 ± 0.508	*p* = 0.325
REBA	0.116 ± 1.128	*p* < 0.001	−0.003 ± 0.208	*p* = 0.373

**Table 7:** Accuracy of REBA score by different methods in nursing care tasks.

Joints	Acc				
	OpenPose	Tsai et al. [23]	Guo et al. [24]	Kanazawa et al. [25]	Ours
Trunk	91.92%	90.34%	92.36%	95.32%	95.65%
Leg-R	81.43%	86.61%	86.42%	88.33%	87.47%
Upper arm-R	71.61%	72.41%	72.98%	75.79%	76.95%
Lower arm-R	47.76%	59.87%	60.14%	62.87%	64.31%
Neck	76.96%	82.86%	87.95%	86.97%	87.96%
Leg-L	82.94%	83.14%	89.76%	91.61%	90.81%
Upper arm-L	80.25%	85.27%	92.31%	91.89%	92.13%
Lower arm-L	84.26%	87.35%	91.14%	95.57%	91.68%
REBA	58.33%	63.29%	76.63%	80.46%	87.34%

## Data Availability

The data presented in this study are available on request from the corresponding author.

## Supplementary Materials

A demo could be found at https://github.com/Nicxhan/Skeleton-compensation-and-correction, accessed on 1 January 2024.
